# Supplementary material for: Development and Validation of a Risk Scoring Tool for Bronchopulmonary Dysplasia in Preterm Infants Based on a Systematic Review and Meta-Analysis
Source: Healthcare (Basel). 2023 Mar 6;11(5):778. doi: 10.3390/healthcare11050778 (PMC10000930; doi:10.3390/healthcare11050778)
Supplement: Supplementary file 1 [file healthcare-11-00778-s001.zip › healthcare-2092449-supplementary.pdf]

SUPPLEMENTARY Materials

Development and validation of a risk scoring tool for bronchopulmonary dysplasia in preterm infants based on a systematic review and meta-analysis

Table S1. Baseline characteristics of the 58 studies.
Table S2. Scoring results of Newcastle Ottawa quality assessment scale.
Table S3. Risk factors of the 58 studies.
Table S4. 24 risk factors include in the systematic review and meta-analysis.
Table S5. Publication bias of risk factors for BPD.
Table S6. Baseline characteristics of preterm infants in validation cohort.
Table S7: A predictive scoring system for BPD in preterm infants.
Table S8: The sensitivity, specificity and Youden index of different cutoff risk scores.
Figure S1: Forest plot: effect of CA on BPD in preterm infants.
Figure S2: Forest plot: effect of GA on BPD in preterm infants.
Figure S3: Forest plot: effect of BW on BPD in preterm infants.
Figure S4: Forest plot: effect of Sex (male) on BPD in preterm infants.
Figure S5: Forest plot: effect of SGA on BPD in preterm infants.
Figure S6: Forest plot: effect of 5 min Apgar score on BPD in preterm infants.
Figure S7: Forest plot: effect of DRI on BPD in preterm infants.
Figure S8: Forest plot: effect of Surfactant on BPD in preterm infants.
Figure S9: Forest plot: effect of RDS on BPD in preterm infants.

This supplementary material has been provided by the authors to give readers additional information about their work.

Table S1. Baseline characteristics of the 58 studies

First author, Year	Country (centers)	Research time	Prospective / Retrospective	Cohort / Case-control	BW (g) & GA (weeks) Mean ± SD / Median (IQR)	Case / Control group	Definition BPD	NOS Score
Abele-Horn 1998	Germany(3)	1993.01 - 1994.12	Prospective	Cohort	934.05 ± 260.7 & 27 ± 1.8	48/49	BPD28 & Chest X-rays	9
Hannaford 1999	Australia(2)	1993.10 - 1994.10 & 1995.01 - 1996.11 & 1996.02 - 1997.06	Prospective	Cohort	832.2 ± 175.7 & 25.8 ± 1.1	38/105	BPD36	9
Korhonen 1999	Finland(1)	1990 - 1994	Retrospective	Cohort	1125.4 ± 242.1 & 28.4 ± 2.2	59/133	BPD28	8
Marshall 1999	USA(13)	1994.01.01 - 1994.12.31	Prospective	Cohort	1100.1 ± 252.9 & 228.9 ± 2.6	224/641	BPD36	9
Grupo Colaborativo Neocosur 2002	South American(11)	1997.10 - 1998.8	Prospective	Cohort	1129 ± 226 & 29.6 ± 3.4	89/296	BPD28	8
Redline 2002	USA(1)	1995 - 1997	Retrospective	Cohort	1023.1 ± 268.7& 27.6 ± 2.67	112/259	BPD36	9
Reiss 2003	Germany(NA)	1990.01 - 1996.12	Retrospective	Cohort	1196.4 ± 367.3 & 28.8 ± 2.1	185/1010	BPD28	8
Antonucci 2004	Italy(1)	1993.01 - 1999.02	Retrospective	Case-control	1148 ± 226 & 30.1 ± 2.7	141/136	BPD28 & Chest X-rays	8
Choi 2005	South Korea(1)	1995.1 - 2000.12	Prospective	Cohort	1168 ± 202 & 29.9 ± 2.2	42/73	BPD28 & BPD36	8
Hentschel 2005	Switzerland(9)	1996 & 2000	Prospective	Cohort	1084.85 ± NA & 29.25 ± NA	100&84/ 550&552	BPD36	8
Tai 2005	China Taiwan(8)	1996.01.01 - 2000.12.31	Retrospective	Cohort	1173.7 ± 226 & 29.4 ± 2.9	538/1225	BPD36	8
Henderson-Smart 2006	Australia& New Zealand(25)	1998 - 2001	Prospective	Cohort	1235(960 - 1510) & 29(27 - 30)	2710/8743	BPD36	8
Tapia 2006	South American(16)	2000.10 - 2003.12	Prospective	Cohort	1210 ± 243 & 30 ± 2.6	446/1379	BPD28	8
Ameenudeen 2007	Malaysia(1)	2003.04.01 - 2005.8.31	Prospective	Cohort	1141 ± 237 & 29.7 ± 2.7	36/208	BPD36& Chest X-rays	9
Bhering 2007	Brazil(1)	1998.01 - 2001.07 & 2001.08 - 2003.07	Retrospective & Prospective	Cohort	1083 ± 237 & 29.1 ± 2.4	68/179	BPD28	8
Honma 2007	Japan(1)	1997.11 - 2001.08	Retrospective	Cohort	1143 ± 326 & 28.7 ± 1.99	39/66	BPD28	7
Demirel 2009	Turkey(1)	2004.01 - 2006.12	Retrospective	Case-control	1214.48 ± 214.97 & 29.46 ± 2.14	56/50	BPD36	8
Guimarães 2010	Portugal(5)	2004.01.01 - 2006.12.31	Prospective	Cohort	1116.9 ± 165.4 & 29.0 ± 1.2	33/223	BPD36& Chest X-rays	8
Gantar 2011	Slovenia(1)	2000.09.01 - 2002.6.30	Prospective	Cohort	1034 ± 236 &	25/90	BPD36	8

					27.2 ± 1.5			
Gortner 2011	Europe(NA)	2003	Prospective	Cohort	1250(370 - 3150) & 30(23 - 31) **Median (range)**	677/3508	BPD36	8
Gagliardi 2011	Italy(14)	1999 - 2002	Prospective	Cohort	1120(410 - 1499) & 29(23 - 31) **Median (range)**	196/1064	BPD36	9
Zhang 2011	China(1)	1999.01 - 2009.01	Retrospective	Case-control	1248 ± 195 & 30.20 ± 2.17	56/60	BPD28	8
Löfqvist 2012	Sweden(2)	1999.12 - 2002.04 & 2005.01 - 2007.05	Retrospective	Cohort	991 ± 337 & 27.2 ± 2.2	50/58	BPD36	8
O'Shea 2012	Australia(NA)	1991 - 1992 & 1997 & 2005	Prospective	Cohort	862 ± 170 & 26.6 ± 2.1	332/419	BPD36	9
Rojas 2012	Colombia(8)	2004.01.01 - 2006.12.31	Prospective	Case-control	1271.2 ± 283.3 & 29.2 ± 1.3	64/148	BPD36	8
Klinger 2013	Israel(28)	2000 - 2010	Prospective	Cohort	NA	1663/10476	BPD36	8
Shima 2013	Japan(1)	2000.01 - 2009.12	Retrospective	Cohort	1236 ± 323 & 29.0 ± 1.3	53/253	BPD36	8
Yen 2013	China Taiwan(21)	1997 - 2006	Retrospective	Cohort	1183.5 ± 276.8 & 29.3 ± 3.1	2008/3745	BPD36	9
AlShehri 2014	Saudi Arabia(1)	2002.01 - 2011.12	Retrospective	Cohort	1078 ± 176 & 29.3 ± 1.8	266/676	BPD28 & BPD36	8
Gursoy 2015	Turkey(1)	2006 - 2009	Retrospective	Cohort	1218 ± 220 & 29.4 ± 1.9	150/502	BPD28	8
Novitsky 2015	USA(1)	2004.07 - 2009.06	Retrospective	Cohort	1056 ± 285 & 28.1 ± 3.5	182/724	BPD36	8
Sasi 2015	Australia(1)	2002 - 2012	Retrospective	Cohort	1116.7 ± 371.3 & 28.7 ± 1.8	123/336	BPD36	9
Duan 2016	China(1)	2014.02 - 2015.02	Prospective	Cohort	1418 ± 297 & 29 (25 - 31) **Median (range)**	71/172	BPD28 & BPD36	9
Fonseca 2017	Brazil(1)	2011.01 - 2014.10	Prospective & Retrospective	Cohort	1200.8 ± 264.3 & 30.1 ± 2.3	94/229	BPD28	8
Lio 2017	Italy(1)	2009.01 - 2013.06	Prospective	Cohort	909 ± 243 & 27.1 ± 1.5	20/134	BPD36	8
Soliman 2017	Canada(1)	2007.01 - 2010.06	Prospective	Cohort	1186.9 ± 398.8 & 28.8 ± 2.0	79/240	BPD36	8
Jung 2019	South Korea(64)	2013.01.01 - 2015.12.31	Prospective	Cohort	790.4 ± 143.6 & 26.2 ± 1.6	816/618	BPD36	9
Mahallei 2019	Iran(1)	2017.03 - 2018.03	Prospective	Cohort	1100.66 ± 216.65 & 28.56 ± 1.95	33/28	BPD28	8
Malikiwi 2019	Australia(1)	2011.01 - 2013.12	Retrospective	Case-control	789.8 ± 122.8 & 26 ± 1.2	33/33	BPD36	8
Patel 2019	USA(3)	2010.01 - 2013.06	Retrospective	Cohort	1024 (790 - 1233) &	240/358	BPD28 &	9

					28 (26 - 30)		BPD36	
Rocha 2019	Portugal(11)	2015.01.01 - 2016.12.31	Prospective	Cohort	963 ± 256 & 27.6 ± 10.3	119/375	BPD36	8
Ryan 2019	USA(11)	2011 - 2013	Prospective	Cohort	913.6 ± 233.4 & 26.7 ± 1.4	298/409	BPD36	8
Tagliaferro 2019	USA(1)	2005.01 - 2015.12	Prospective	Cohort	809.2 ± 231.2 & 25.7 ± 1.6	354/679	BPD36	8
Zhou 2019	China(1)	2011 - 2014	Prospective	Cohort	1380.3 ± 249.4 & 30.43 ± 1.44	22/104	BPD28 & BPD36	8
Chen 2020	China(1)	2016.01 - 2018.07	Retrospective	Cohort	910 ± 199.25 & 26.4 ± 1.5	62/141	BPD36	8
Cokyaman 2020	Turkey(1)	2006.01.01 - 2008.12.31	Retrospective	Cohort	1212 ± 211 & 31.1 ± 2.6	139/551	BPD28	8
Shin 2020	South Korea(NA)	2013.01 - 2014.12	Retrospective	Cohort	1010.6 ± 255.9 & 27.3 ± 1.8	764/1603	BPD36	9
Cai 2021	China(1)	2017.01 - 2019.09	Retrospective	Case-control	1209.6 ± 199.7 & 29.95 ± 2.15	63/388	BPD28	8
Ebrahimi 2021	Netherland(1)	2009.01 - 2015.12	Retrospective	Cohort	936.9 ± 248.3 & 27.2 ± 2.0	79/130	BPD28	8
EI Faleh 2021	Switzerland(9)	2009.01.01 - 2010.12.31	Prospective	Cohort	1230.7 ± 337.7& 29.6 ± 1.8	138/1087	BPD36	8
Jassem-Bobowicz 2021	Switzerland(1)	2013.01 - 2017.03	Retrospective	Cohort	1214.9 ± NA & 29.0 ± NA	127/151	BPD28	8
Li 2021	China(33)	2017 - 2018	Retrospective	Cohort	938 ± 162 & 27.7 ± 1.8	181/446	BPD36	7
Lu 2021	China(1)	2018.01 - 2020.12	Retrospective	Case-control	1241.8 ± 161.5 & 29.9 ± 1.9	79/207	BPD28	8
Ming 2021	China(1)	2018.08.01 - 2020.08.31	Retrospective	Case-control	1460 (1231.3, 1687.5) & 30.7 (29.6, 31.4)	135/229	BPD36	9
Nakashima 2021	Japan(218)	2003.01.01 - 2016.12.31	Retrospective	Cohort	746 (604 - 907) & 25.9 (24.4 - 27.0)	7792/9334	BPD28 & BPD36	8
Shim 2021	South Korea(NA)	2013.01.01 - 2016.12.31	Retrospective	Cohort	1118.5 ± 263.7 & 28.67 ± 2.6	2583/2017	BPD36	8
Sucasas Alonso 2022	Spain(1)	2013.01.01 - 2020.08.30	Retrospective	Cohort	1142.1 ± 255.5 & 29.5 ± 2.1	58/144	BPD28 &BPD36	8
Wang 2022	China(1)	2015.01.01 - 2019.08.31	Retrospective	Case-control	1227.4 ± 199.6 & 28.3 ± 2.9	64/70	BPD28	9

Note: NA, not available; GA, gestational age; BW, birth weight; Mean ± SD, Mean ± standard deviation; IQR, interquartile range; BPD, bronchopulmonary dysplasia; BPD28, bronchopulmonary dysplasia defined as oxygen dependency at 28 days of life; BPD36, bronchopulmonary dysplasia defined as oxygen dependency at 36 weeks postmenstrual age; Chest X-rays, characteristic chest radiographic findings.

Table S2. Scoring results of Newcastle Ottawa quality assessment scale

First author, year	Selection	Compar-ability	Outcome	Total scores	Reasons for downgrade
Abele-Horn 1998	* * * *	* *	* * *	9	
Hannaford 1999	* * * *	* *	* * *	9	
Korhonen 1999	* * * *	*	* * *	8	No adjustment for confounders
Marshall 1999	* * * *	* *	* * *	9	
Grupo Colaborativo Neocosur 2002	* * * *	*	* * *	8	No adjustment for confounders
Redline 2002	* * * *	* *	* * *	9	
Reiss 2003	* * * *	*	* * *	8	No adjustment for confounders
Antonucci 2004	* * * *	*	* * *	8	No adjustment for confounders
Choi 2005	* * * *	*	* * *	8	No adjustment for confounders
Hentschel 2005	* * * *	*	* * *	8	No adjustment for confounders
Tai 2005	* * * *	*	* * *	8	No adjustment for confounders
Henderson-Smart 2006	* * * *	*	* * *	8	No adjustment for confounders
Tapia 2006	* * * *	*	* * *	8	No adjustment for confounders
Ameenudeen 2007	* * * *	* *	* * *	9	
Bhering 2007	* * * *	*	* * *	8	No adjustment for confounders
Honma 2007	* * *	*	* * *	7	Non-representative population (All multiple-birth infants excluded), No adjustment for confounders
Demirel 2009	* * * *	*	* * *	8	No adjustment for confounders
Guimarães 2010	* * * *	*	* * *	8	No adjustment for confounders
Gantar 2011	* * * *	*	* * *	8	No adjustment for confounders
Gortner 2011	* * * *	*	* * *	8	No adjustment for confounders
Gagliardi 2011	* * * *	* *	* * *	9	
Zhang 2011	* * * *	*	* * *	8	No adjustment for confounders
Löfqvist 2012	* * * *	*	* * *	8	No adjustment for confounders
O'Shea 2012	* * * *	* *	* * *	9	
Rojas 2012	* * * *	*	* * *	8	No adjustment for confounders
Klinger 2013	* * * *	*	* * *	8	No adjustment for confounders
Shima 2013	* * * *	*	* * *	8	No adjustment for confounders
Yen 2013	* * * *	* *	* * *	9	
AlShehri 2014	* * * *	*	* * *	8	No adjustment for confounders
Gursoy 2015	* * * *	*	* * *	8	No adjustment for confounders
Novitsky 2015	* * * *	*	* * *	8	No adjustment for confounders
Sasi 2015	* * * *	* *	* * *	9	
Duan 2016	* * * *	* *	* * *	9	
Fonseca 2017	* * * *	*	* * *	8	No adjustment for confounders
Lio 2017	* * *	*	* * *	8	No adjustment for confounders
Soliman 2017	* * *	*	* * *	8	No adjustment for confounders

Jung 2019	* * * *	* *	* * *	9	
Mahallei 2019	* * *	* *	* * *	8	Non-representative population (all infants intubated)
Malikiwi 2019	* * * *	*	* * *	8	No adjustment for confounders
Patel 2019	* * * *	* *	* * *	9	
Rocha 2019	* * * *	*	* * *	8	No adjustment for confounders
Ryan 2019	* * *	*	* * *	8	No adjustment for confounders
Tagliaferro 2019	* * * *	*	* * *	8	No adjustment for confounders
Zhou 2019	* * * *	*	* * *	8	No adjustment for confounders
Chen 2020	* * * *	*	* * *	8	No adjustment for confounders
Cokyaman 2020	* * * *	*	* * *	8	No adjustment for confounders
Shin 2020	* * * *	* *	* * *	9	
Cai 2021	* * * *	*	* * *	8	No adjustment for confounders
Ebrahimi 2021	* * *	* *	* * *	8	Non-representative population (all infants with one or more late-onset sepsis)
EI Faleh 2021	* * * *	*	* * *	8	No adjustment for confounders
Jassem-Bobowicz 2021	* * * *	*	* * *	8	No adjustment for confounders
Li 2021	* * * *	*	* *	7	No adjustment for confounders; Loss to follow-up
Lu 2021	* * * *	*	* * *	8	No adjustment for confounders
Ming 2021	* * * *	* *	* * *	9	
Nakashima 2021	* * * *	*	* * *	8	No adjustment for confounders
Shim 2021	* * * *	*	* * *	8	No adjustment for confounders
Sucasas Alonso 2022	* * * *	*	* * *	8	No adjustment for confounders
Wang 2022	* * * *	* *	* * *	9	

Table S3. Risk factors of the 58 studies

First author, year	MHD	CA	Antenatal steroids	PROM	GA	BW	Sex (male)	SGA	Caesarean section	5 min Apgar score	DRI	Neonatal asphyxia	MV	Days of MV	MV > 7 days	Postnatal steroids	Surfactant	PDA	RDS	ROP	Sepsis	IVH	NEC	Pulmonary air leak
Abele-Horn 1998		●			●	●			●					●		●	●		●		●			
Hannaford 1999					●																			
Korhonen 1999							●	●																
Marshall 1999						●											●							
Grupo Colaborativo Neocosur 2002					●	●											●						●	
Redline 2002					●													●						●
Reiss 2003					●		●	●							●		●				●			
Antonucci 2004															●			●	●					
Choi 2005		●			●		●			●								●	●		●			●
Hentschel 2005													●				●	●						
Tai 2005					●	●			●									●			●			
Henderson-Smart 2006							●																	
Tapia 2006					●	●	●										●	●					●	●
Ameenudeen 2007		●					●											●						
Bhering 2007	●								●		●							●	●					
Honma 2007		●		●	●	●																		
Demirel 2009													●				●		●					
Guimarães 2010						●															●			
Gantar 2011							●				●						●	●						●
Gortner 2011	●						●	●																
Gagliardi 2011							●	●							●									
Zhang 2011		●																						
Löfqvist 2012					●	●	●			●			●	●		●		●		●		●		
O'Shea 2012					●	●	●																	

Rojas 2012				●													●	●			●		
Klinger 2013	●						●	●															
Shima 2013								●										●					
Yen 2013	●				●	●	●												●		●		
Alshehri 2014			●										●					●		●		●	●
Gursoy 2015							●												●			●	
Novitsky 2015		●																					
Sasi 2015													●										
Duan 2016																	●	●		●			
Fonseca 2017					●														●				
Lio 2017						●		●		●				●							●		
Soliman 2017																	●				●		
Jung 2019							●																
Mahallei 2019					●																		
Malikiwi 2019					●																		
Patel 2019					●	●		●															
Rocha 2019	●																						
Ryan 2019			●		●						●												
Tagliaferro 2019	●				●			●										●			●		
Zhou 2019				●			●									●							
Chen 2019					●					●	●						●						
Cokyaman 2020							●											●	●				
Shin 2020		●			●		●	●											●				
Cai 2021												●						●	●		●		
Ebrahimi 2021													●			●	●						
El Faleh 2021																	●	●					
Bobowicz 2021		●							●		●						●					●	●
Li 2021			●				●				●												
Lu 2021												●											
Ming 2021				●								●		●				●	●		●		

Nakashima 2021		●			●		●				●	●
Shim 2021	●		●	●		●			●			
Sucasas Alonso 2022							●		●	●		●
Wang 2022				●				●				

Note: MHD, maternal hypertensive disorders; CA, Chorioamnionitis; PROM, premature rupture of membrane; GA, gestational age; BW, birth weight; SGA, small for gestational age; DRI, delivery room intubation; MV, mechanical ventilation; PDA, patent ductus arteriosus; RDS, respiratory distress syndrome; ROP, retinopathy of prematurity; IVH, intraventricular hemorrhage; NEC, necrotizing enterocolitis.

Table S4. 24 risk factors include in the systematic review and meta-analysis

Meta-analysis	No. of Studies	OR	95% CI	*p* Value	Heterogeneity	
					I^2, %	*p* Value
MHD	7	1.09	[0.76,1.58]	0.634	84.2	0.000
CA	9	2.82	[1.92,4.15]	0.000	82.8	0.000
Antenatal steroids	3	0.76	[0.39,1.49]	0.428	88.5	0.000
PROM	4	1.32	[0.67,2.59]	0.418	87.1	0.000
GA	21	0.64	[0.58,0.70]	0.000	95.1	0.000
BW	12	0.80	[0.77,0.83]	0.000	55.1	0.011
Sex (male)	21	1.50	[1.38,1.63]	0.000	47.6	0.009
SGA	11	2.90	[1.94,4.35]	0.000	93.9	0.000
Caesarean section	4	1.00	[0.47,2.12]	0.990	86.4	0.000
5 min Apgar score	5	0.71	[0.64,0.78]	0.000	0	0.524
DRI	8	2.66	[2.02,3.50]	0.000	66.3	0.004
Neonatal asphyxia	3	2.08	[1.42,3.04]	0.000	0	0.488
MV	7	3.28	[2.14,5.03]	0.000	63.9	0.011
Days of MV	4	1.10	[1.08,1.12]	0.000	23.1	0.272
MV>7 days	3	19.50	[10.82,35.14]	0.000	0	0.404
Postnatal steroids	4	5.93	[3.67,9.61]	0.000	45.5	0.138
Surfactant	17	3.42	[2.43,4.83]	0.000	86.3	0.000
PDA	20	3.09	[2.31,4.13]	0.000	90.4	0.000
RDS	13	4.47	[2.84,7.04]	0.000	95.1	0.000
ROP	3	3.56	[0.94,13.49]	0.061	92	0.000
Sepsis	14	2.07	[1.67,2.57]	0.000	83.4	0.000
IVH	4	2.75	[1.90,3.97]	0.000	0	0.544
NEC	4	2.11	[1.29,3.47]	0.000	78.0	0.003
Pulmonary air leak	4	3.15	[1.25,7.92]	0.015	79.4	0.002

Note: OR, Odds ratio; CI, confidence interval; MHD, maternal hypertensive disorders; CA, Chorioamnionitis; PROM, premature rupture of membrane; GA, gestational age; BW, birth weight; SGA, small for gestational age; DRI, delivery room intubation; MV, mechanical ventilation; PDA, patent ductus arteriosus; RDS, respiratory distress syndrome; ROP, retinopathy of prematurity; IVH, intraventricular hemorrhage; NEC, necrotizing enterocolitis.

Table S5: Publication bias of risk factors for BPD

Risk factors	Begg's test		Egger's test	
	**Z**	**Pr>|z|**	**t**	***p***
CA	2.19	0.029*	8.39	0.000*
GA	1.18	0.239	-2.27	0.035*
BW	1.30	0.193	-1.38	0.199
Sex (male)	3.11	0.002*	2.40	0.027*
SGA	0.00	1.000	1.26	0.241
5 min Apgar score	0.24	0.806	-1.34	0.273
DRI	0.87	0.386	4.33	0.005*
Neonatal asphyxia	1.04	0.296	11.29	0.056
MV	1.80	0.072	4.75	0.005*
Days of MV	1.02	0.308	5.46	0.032*
MV>7 days	1.04	0.296	3.94	0.158
Postnatal steroids	1.70	0.089	3.98	0.058
Surfactant	1.85	0.064	4.65	0.000*
PDA	2.17	0.030*	5.74	0.000*
RDS	0.67	0.502	3.35	0.006*
Sepsis	1.97	0.049*	5.27	0.000*
IVH	1.70	0.089	3.81	0.062
NEC	1.70	0.089	8.74	0.013*
Pulmonary air leak	1.02	0.308	12.57	0.006*

Note: CA, Chorioamnionitis; GA, gestational age; BW, birth weight; SGA, small for gestational age; DRI, delivery room intubation; MV, mechanical ventilation; PDA, patent ductus arteriosus; RDS, respiratory distress syndrome; IVH, intraventricular hemorrhage; NEC, necrotizing enterocolitis.

*, $p < 0.05$.

Table S6: Baseline characteristics of preterm infants in validation cohort

Variables	Total（N = 767）	BPD Yes(N = 185)	BPD No(N = 582)	*p* value
MHD [n(%)]				0.814
Yes	112(14.6)	28(15.1)	84(14.4)	
No	655(85.4)	157(84.9)	498(85.6)	
CA [n(%)]				0.000
Yes	119(15.5)	69(37.3)	50(8.6)	
No	648(84.5)	116(62.7)	532(91.4)	
Antenatal steroids [n(%)]				0.917
Yes	383(49.9)	93(50.3)	290(49.8)	
No	384(50.1)	92(49.7)	292(50.2)	
PROM [n(%)]				0.473
Yes	257(33.5)	66(35.7)	191(32.8)	
No	510(66.5)	119(64.3)	391(67.2)	
GA (weeks)	30.14 ± 1.79	28.68 ± 1.74	30.60 ± 1.54	0.000
BW (g)	1306.04 ± 314.28	1053.06 ± 239.83	1386.46 ± 291.80	0.000
Sex[n(%)]				0.003
Male	399(52.0)	114(61.6)	285(49.0)	
Female	368(48.0)	71(38.4)	297(51.0)	
SGA[n(%)]				0.000
Yes	232(30.2)	83(44.9)	149(25.6)	
No	535(69.8)	102(55.1)	433(74.4)	
Caesarean section [n(%)]				0.000
Yes	543(70.8)	108(58.4)	435(74.7)	
No	224(29.2)	77(41.6)	147(25.3)	
5 min Apgar score	8.63 ± 1.89	6.50 ± 1.87	9.31 ± 1.30	0.000
DRI [n(%)]				0.000
Yes	190(24.8)	95(51.4)	95(16.3)	
No	577(75.2)	90(48.6)	487(83.7)	
Neonatal asphyxia [n(%)]				0.000
Yes	357(46.5)	128(69.2)	229(39.3)	
No	410(53.5)	57(30.8)	353(60.7)	
Days of MV (days)	2.91 ± 7.26	7.77 ± 12.84	1.36 ± 2.73	0.000
MV [n(%)]				0.000
Yes	290(37.8)	119(64.3)	171(29.4)	
No	477(62.2)	66(35.7)	411(70.6)	
MV > 7 days				0.000
Yes	89(11.6)	62(33.5)	27(4.6)	
No	678(88.4)	123(66.5)	555(95.4)	
Postnatal steroids [n(%)]				0.000
Yes	153(19.9)	87(47.0)	66(11.3)	
No	614(80.1)	98(53.0)	516(88.7)	
Surfactant [n(%)]				0.000

Yes	334(43.5)	126(68.1)	208(35.7)	
No	433(56.5)	59(31.9)	374(64.3)	
PDA[n(%)]				0.000
Yes	141(18.4)	64(34.6)	77(13.2)	
No	626(81.6)	121(65.4)	505(86.8)	
RDS[n(%)]				0.000
Yes	583(76.0)	177(95.7)	406(69.8)	
No	184(24.0)	8(4.3)	176(30.2)	
ROP[n(%)]				0.000
Yes	143(18.6)	74(40.0)	69(11.9)	
No	624(81.4)	111(60.0)	513(88.1)	
Sepsis [n(%)]				0.000
Yes	215(28.0)	78(42.2)	137(23.5)	
No	552(72.0)	107(57.8)	445(76.5)	
IVH [n(%)]				0.385
Yes	14(1.8)	2(1.1)	12(2.1)	
No	753(98.2)	183(98.9)	570(97.9)	
NEC [n(%)]				0.625
Yes	56(7.3)	12(6.5)	44(7.6)	
No	711(92.7)	173(93.5)	538(92.4)	
Pulmonary air leak[n(%)]				0.055
Yes	58(7.6)	20(10.8)	38(6.5)	
No	709(92.4)	165(89.2)	544(93.5)	

Note: MHD, maternal hypertensive disorders; CA, Chorioamnionitis; PROM, premature rupture of membrane; GA, gestational age; BW, birth weight; SGA, small for gestational age; DRI, delivery room intubation; MV, mechanical ventilation; PDA, patent ductus arteriosus; RDS, respiratory distress syndrome; ROP, retinopathy of prematurity; IVH, intraventricular hemorrhage; NEC, necrotizing enterocolitis.

Table S7: A predictive scoring tool for BPD in preterm infants.

BPD scoring tool		
**Risk factors**	**Category**	**Point**
CA	No	0
	Yes	5
GA(weeks)	≥ 32	0
	24 - 31^{+6}	2 × ⌈32 - GA⌉
	< 24	18
BW(g)	≥ 1500	0
	500 - 1499	1 × ⌈15 - BW/100⌉
	< 500	11
Sex	female	0
	male	2
SGA	No	0
	Yes	6
5 min Apgar score	≥ 8	0
	3 - 7	1 × (8 - 5 min Apgar score)
	≤ 2	6
DRI	No	0
	Yes	4
Surfactant	No	0
	Yes	5
RDS	No	0
	Yes	7

Note: BPD, bronchopulmonary dysplasia; CA, Chorioamnionitis; GA, gestational age; BW, birth weight; SGA, small for gestational age; DRI, delivery room intubation; RDS, respiratory distress syndrome;

"⌈ ⌉" means ceiling function, for example, when BW=1450 g, then ⌈15 - BW/100⌉ = 1, the final BW gets 1 points.

Table S8: The sensitivity, specificity and Youden index of different cutoff risk scores

Cut-off value	Sensitivity	Specificity	Youden index
10.5	0.995	0.256	0.251
11.5	0.995	0.306	0.301
12.5	0.995	0.342	0.337
13.5	0.995	0.381	0.376
14.5	0.973	0.443	0.416
15.5	0.962	0.473	0.435
16.5	0.951	0.526	0.477
17.5	0.946	0.562	0.508
18.5	0.935	0.603	0.538
19.5	0.919	0.656	0.575
20.5	0.908	0.704	0.612
21.5	0.903	0.741	0.644
22.5	0.897	0.765	0.662
23.5	0.897	0.796	0.693
24.5	0.897	0.837	0.734
25.5	0.897	0.873	0.770*
26.5	0.811	0.887	0.698
27.5	0.703	0.904	0.607
28.5	0.643	0.924	0.567
29.5	0.546	0.936	0.482
30.5	0.497	0.947	0.444
31.5	0.449	0.95	0.399
32.5	0.416	0.964	0.38
33.5	0.384	0.974	0.358
34.5	0.341	0.978	0.319
35.5	0.303	0.978	0.281
36.5	0.259	0.979	0.238
37.5	0.232	0.983	0.215
38.5	0.205	0.99	0.195
39.5	0.205	0.995	0.2
40.5	0.173	0.995	0.168

Note: *, Optimal cutoff point.

Figure S1: CA

A

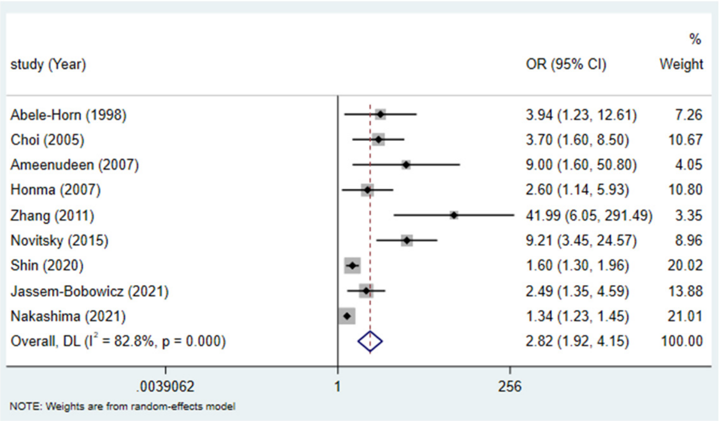

B

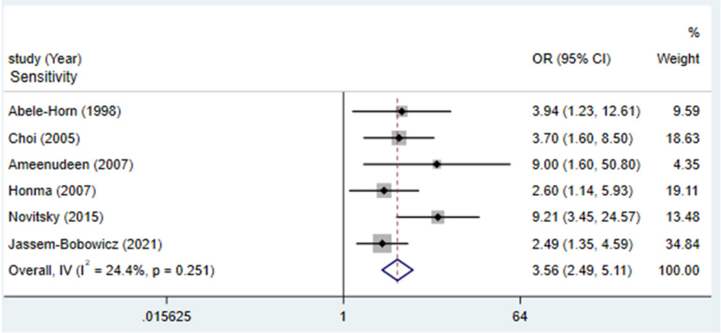

Figure S1 - A. Forest plot: effect of CA on BPD in preterm infants;
Figure S1 - B. Sensitivity analysis: effect of CA on BPD in preterm infants.

Figure S2: GA

A

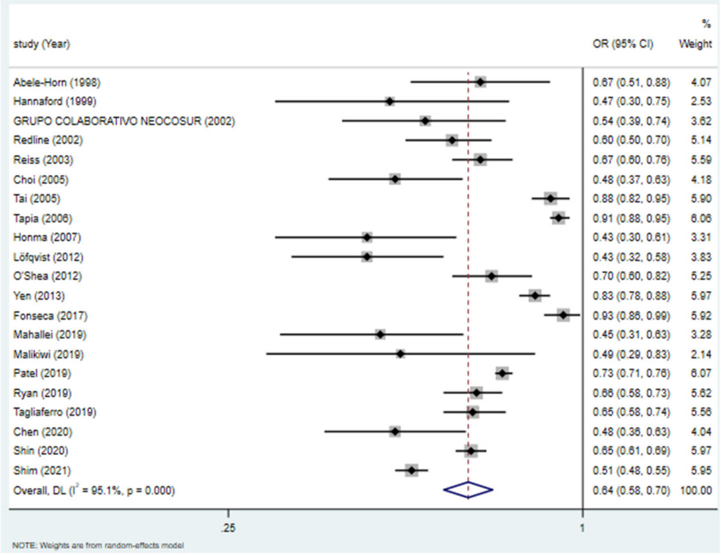

B

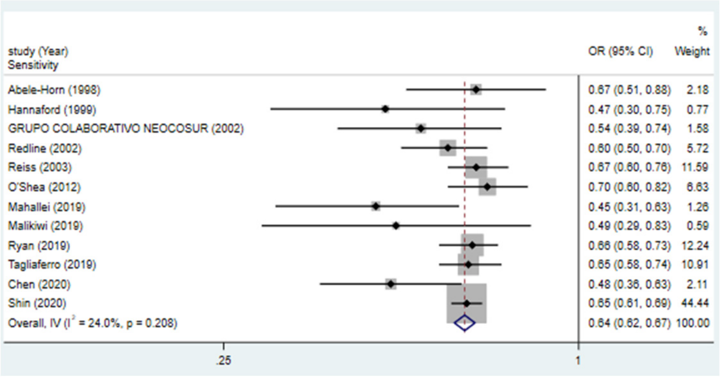

Figure S2 - A. Forest plot: effect of GA on BPD in preterm infants;
Figure S2 - B. Sensitivity analysis: effect of GA (per 1 week increase) on BPD in preterm infants.

Figure S3: BW

A

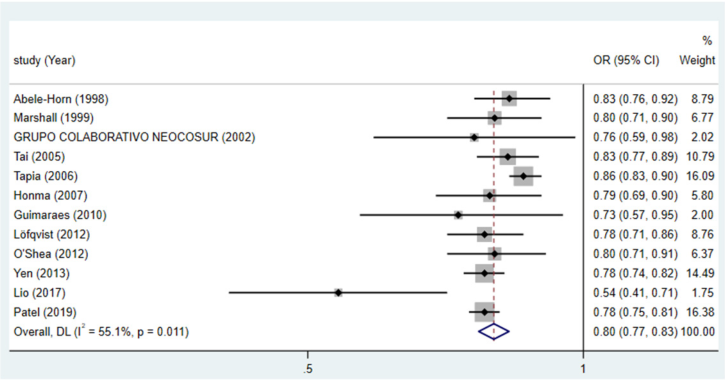

B

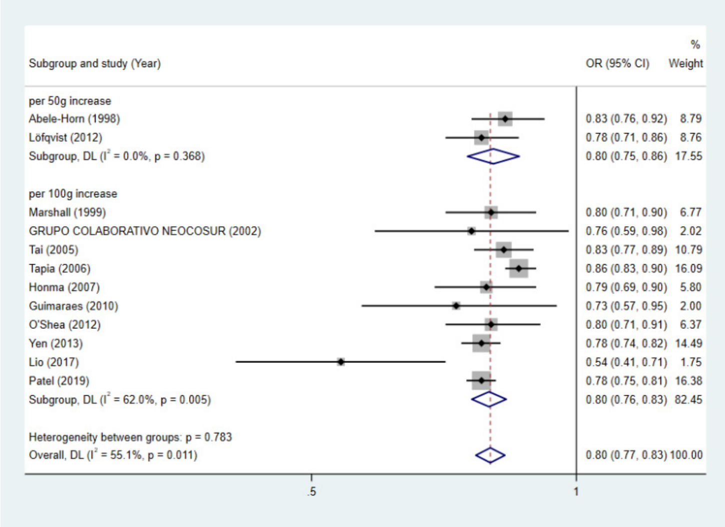

C

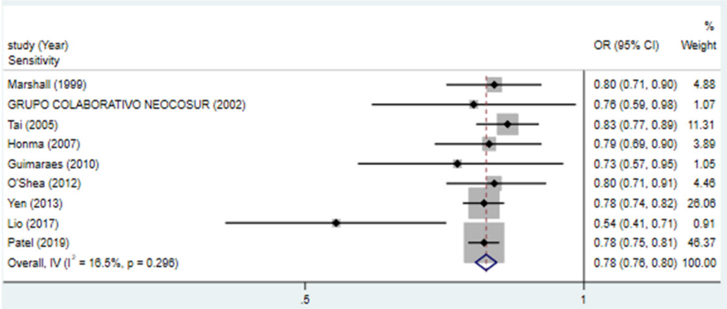

Figure S3 - A. Forest plot: effect of BW on BPD in preterm infants;
Figure S3 - B. Subgroup analysis: effect of BW on BPD in preterm infants;
Figure S3 - C. Sensitivity analysis: effect of BW (per 100 g increase) on BPD in preterm infants.

Figure S4: Sex (male)

A

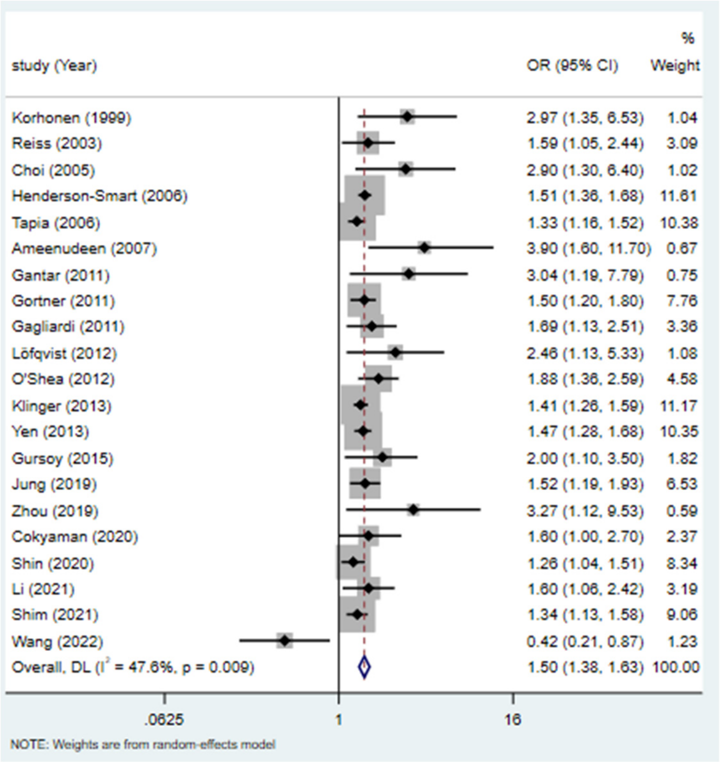

B

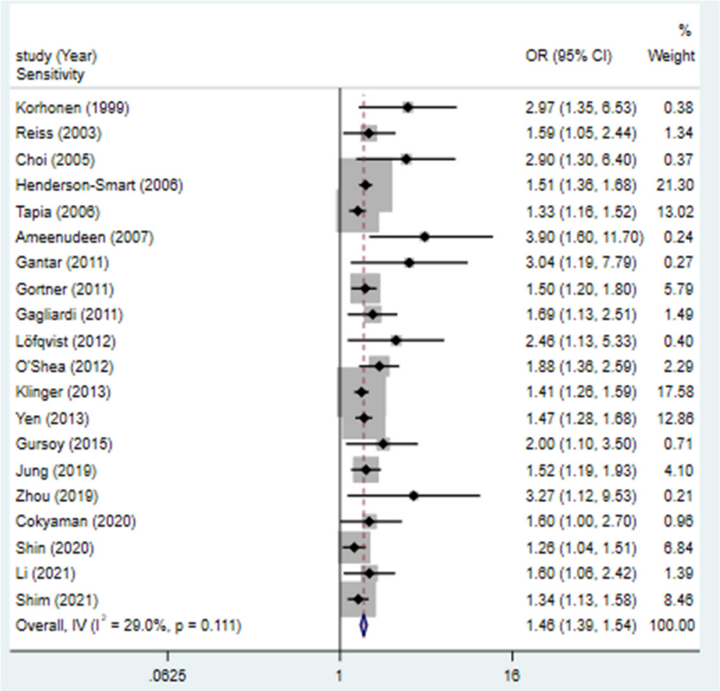

Figure S4 - A. Forest plot: effect of sex on BPD in preterm infants;
Figure S4 - B. Sensitivity analysis: effect of sex on BPD in preterm infants.

Figure S5: SGA

A

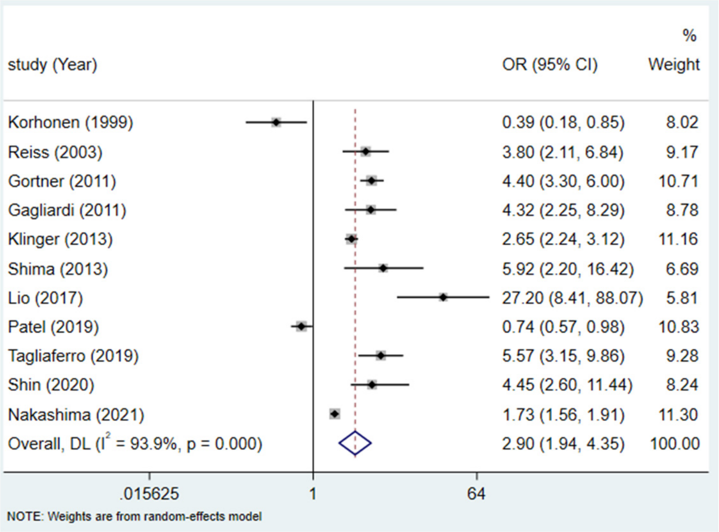

B

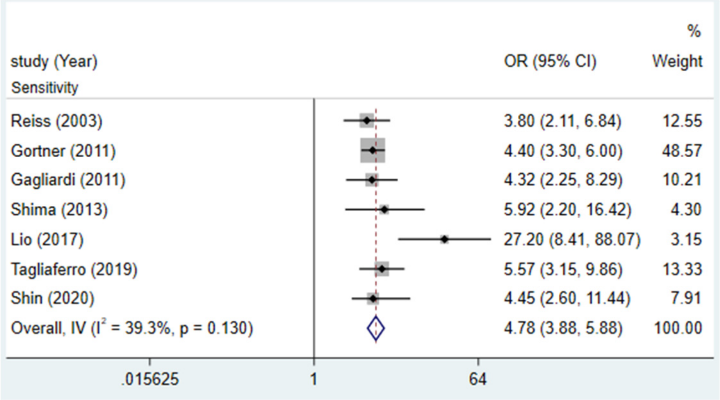

Figure S5 - A. Forest plot: effect of SGA on BPD in preterm infants;
Figure S5 - B. Sensitivity analysis: effect of SGA on BPD in preterm infants.

Figure S6: **5 min Apgar score**

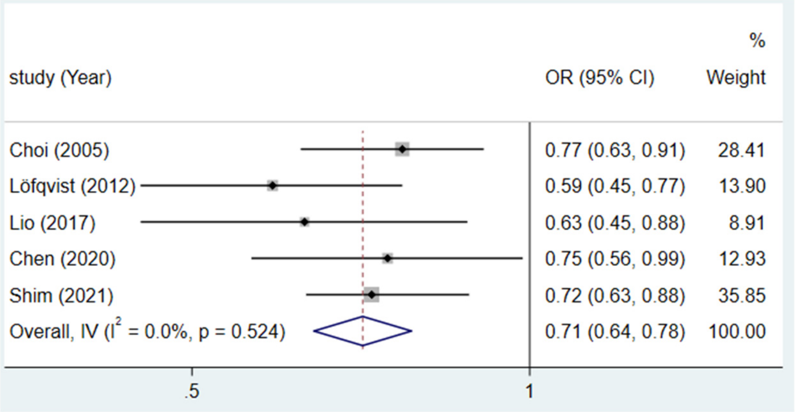

Figure S6. Forest plot: effect of 5 min Apgar score (per 1 point increase) on BPD in preterm infants.

Figure S7: **DRI**

A

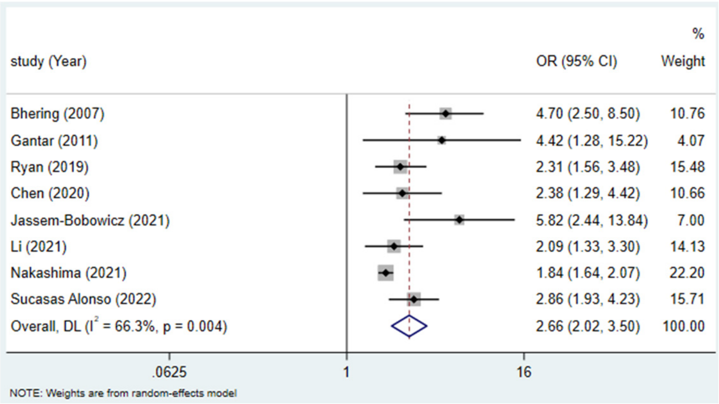

B

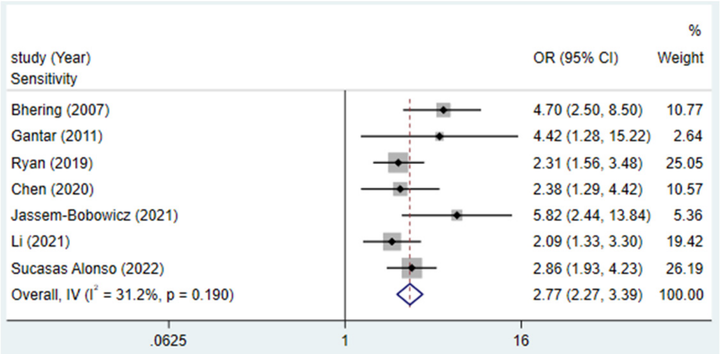

Figure S7 - A. Forest plot: effect of DRI on BPD in preterm infants;
Figure S7 - B. Sensitivity analysis: effect of DRI on BPD in preterm infants.

Figure S8: Surfactant

A

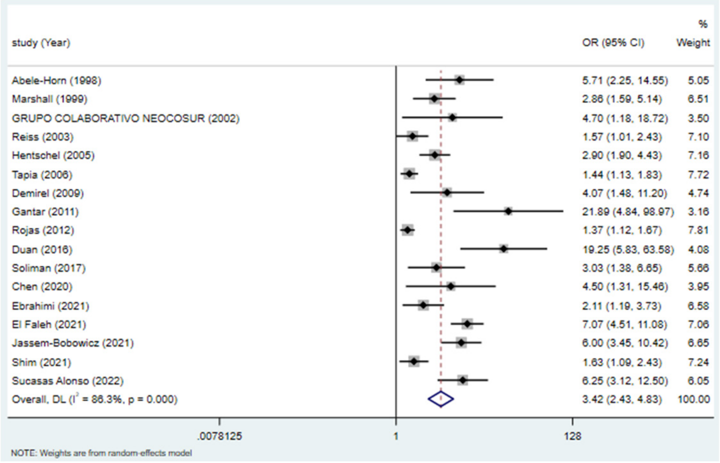

B

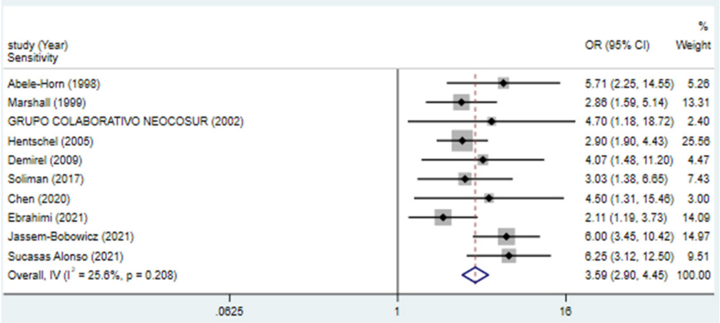

Figure S8 - A. Forest plot: effect of Surfactant on BPD in preterm infants;
Figure S8 - B. Sensitivity analysis: effect of Surfactant on BPD in preterm infants.

Figure S9: RDS

A

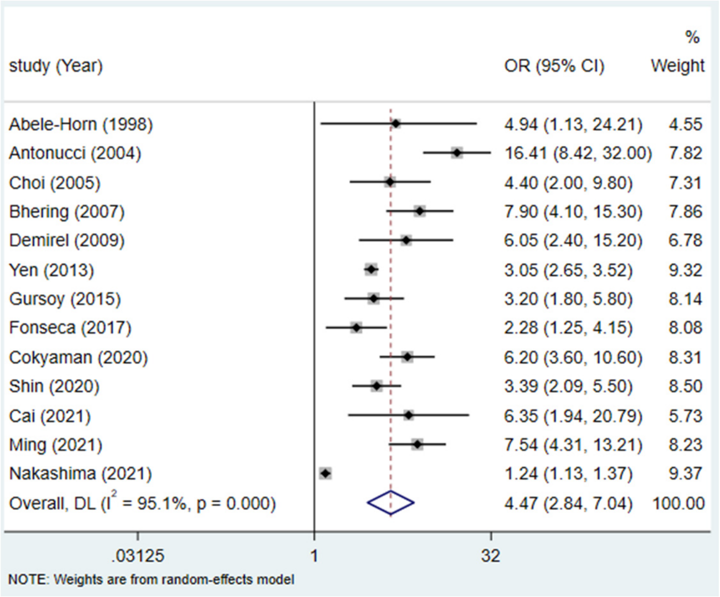

B

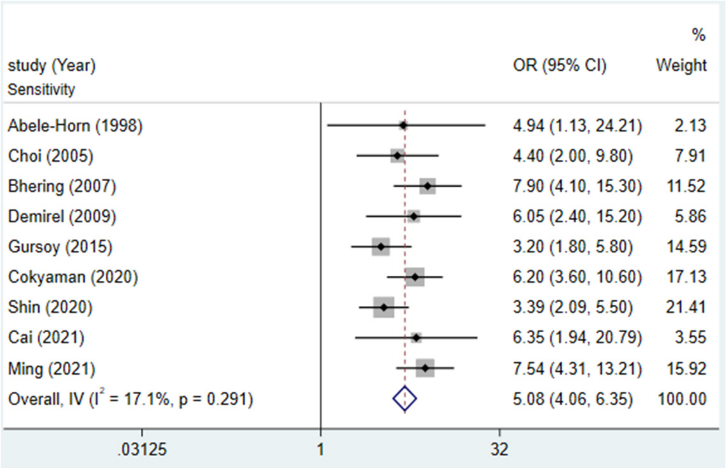

Figure S9 - A. Forest plot: effect of RDS on BPD in preterm infants;
Figure S9 - B. Sensitivity analysis: effect of RDS on BPD in preterm infants.

Note: BPD, bronchopulmonary dysplasia; CA, Chorioamnionitis; GA, gestational age; BW, birth weight; SGA, small for gestational age; DRI, delivery room intubation; RDS, respiratory distress syndrome.
